# Clinical Features and Outcome of the Guillain–Barre Syndrome: A Single-Center 11-Year Experience

**DOI:** 10.3389/fneur.2022.856091

**Published:** 2022-06-29

**Authors:** Federica Ginanneschi, Fabio Giannini, Francesco Sicurelli, Carla Battisti, Giorgio Capoccitti, Sabina Bartalini, Andrea Mignarri, Nila Volpi, David Cioncoloni, Laura Franci, Nicola De Stefano, Alessandro Rossi

**Affiliations:** ^1^Neurology and Clinical Neurophysiology Unit, Department of Medical, Surgical and Neurological Sciences, University of Siena, Siena, Italy; ^2^Clinical Neurology and Neurometabolic Unit, Department of Medical, Surgical and Neurological Sciences, University of Siena, Siena, Italy; ^3^U.O.P. Professioni Della Riabilitazione, AOUS, Siena, Italy

**Keywords:** AMAN, AIDP, electrophysiology, epidemiology, infection, mechanical ventilation

## Abstract

**Background:**

Clinical presentation, electrophysiological subtype, and outcome of the Guillain–Barre' Syndrome (GBS) may differ between patients from different geographical regions. This study aims to assess clinical–neurophysiological features of an adult, Italian GBS cohort over 11 years.

**Methods:**

Retrospective (from 1 January 2011 to 31 December 2021) analysis was carried out on patients admitted to the Siena University Hospital who fulfilled the GBS diagnostic criteria. Demographic data, clinical characteristics, treatment, need of mechanical ventilation (MV), laboratory and electrophysiological tests, preceding infections/vaccination/other conditions, and comorbidities were collected for each patient.

**Results:**

A total of 84 patients (51 men, median age of 61 years), were identified. GBS subtype was classified as acute inflammatory demyelinating polyneuropathy (AIDP) in the 66.6% of patients, acute motor/sensory axonal neuropathy (AMAN/AMSAN) in 20.2%, and the Miller Fisher syndrome in 5 (5.9%). Flu syndrome and gastrointestinal infection were the most common preceding conditions. In total, five (5.9%) subjects had concomitant cytomegalovirus (CMV) infection. Cranial nerve involvement occurred in 34.5% of subjects. Differences between the axonal and AIDP forms of GBS concerned the presence of anti-ganglioside antibodies. In total, seven (8.33%) patients required MV.

**Discussion:**

The epidemiological and clinical characteristics of GBS in different countries are constantly evolving, especially in relation to environmental changes. This study provides updated clinical-epidemiological information in an Italian cohort.

## Introduction

The Guillain–Barré syndrome (GBS) is a rare, but potentially fatal, immune-mediated disease of the peripheral nerves and nerve roots that is usually triggered by infections. The annual, worldwide incidence has been reported as 1.1–1.8 cases per 100,000 people per year (1). The annual incidence rate in Italy is 1.84 per 100,000, among the highest in western countries (2).

Recent evidence support GBS as a spectrum disorder, with regional variations and significant heterogeneity (3, 4). The main forms are acute inflammatory demyelinating polyneuropathy (AIDP), acute motor axonal neuropathy (AMAN), acute sensorimotor axonal neuropathy (AMSAN), and the Miller Fisher syndrome (MFS).

Factors related to geography have a major influence on preceding factors, clinical phenotype, disease severity, electrophysiological subtype, and outcome of GBS (4).

The clinical spectrum ranges from mild to severe symptoms, rapidly progressing weakness. At the most severe end of the spectrum, up to 30% of the patients develop tetraparalysis and respiratory failure, requiring mechanical ventilation (MV) (5). Cranial nerve involvement is a predictor for MV (6, 7), with patients with AIDP more at risk for MV than those with AMAN/AMSAN (8, 9).

Approximately 40–70% of patients with GBS have a previous infectious, whose nature can influence the clinical phenotype and prognosis, and also the electrophysiological subtype. *Campylobacter jejuni* and cytomegalocirus (CMV) are the most commonly isolated pathogens in GBS (10); the former accounts for the pathogenesis of AMAN, the latter mainly for AIDP.

Global variation in infection burden may at least in part explain the regional differences in clinical presentation and subtype of GBS. In Europe and North America, AIDP accounts for up to 90% of cases, whereas axonal GBS is more frequent in Asian and South American countries. Miller Fisher syndrome seems to be more common in Asia than in Europe/Americas (4). Epidemiological differences are also likely to exist within countries. For example, although in Italy axonal forms account for more than 15% of cases (2), the reported local incidence varies between 35% (11) and 4.11% (12).

The present study aims to assess the clinical-epidemiological features of an Italian GBS cohort evaluated at the Siena University Hospital over a period of 11-years.

## Materials and Methods

### Patients

The study was carried out on the medical records of patients who fulfilled the diagnostic criteria of GBS, admitted to the Neurological Units of the Siena University Hospital from 1 January 2011 to 31 December 2021. The patients came principally from Province of Siena and neighboring areas. Medical records were reviewed retrospectively. All the patients were 18 years and older, with the first occurrence of GBS were included. The criteria for diagnosis of GBS were based on the Brighton criteria (13): a natural history compatible with GBS and the absence of an alternative diagnosis. The clinical features considered were: monophasic course and 12 h to 28 days between onset and nadir, bilateral, flaccid weakness of limbs, diminished or absent deep tendon reflex, albumin-cytological dissociation of the cerebrospinal fluid, and nerve conduction study findings.

All the recruited subjects met the 1 or 2° level of diagnostic certainty according to the Brighton criteria (13). Mandatory for the inclusion was the availability of at least two neurographic tests, the first at the time of admission, and the second, after a time interval ranging from a minimum of 8 to a maximum of 14 days. Other exclusion criteria were the lack of clinical and/or laboratory data in patient records.

Demographical data, clinical characteristics, treatment, need of MV, laboratory and electrophysiological tests, preceding infections/vaccination/other conditions, comorbidities, were collected for each patient.

As part of the clinical examination, we recorded symptom onset, muscle strength, sensory disturbances, cranial nerve impairment, the need of MV, laboratory tests, and electrophysiological study. The degree of disability was assessed with the GBS disability score at admission and on discharge (14).

### Laboratory Tests

Laboratory tests included cerebrospinal fluid (CSF) analysis, serology for CMV, the Epstein–Barr virus (EBV), herpes viruses (HSV 1,2), varicella zoster virus (VZV), human immunodeficiency virus (HIV), *Mycoplasma pneumoniae*, Hepatitis B and C, and borrelia burgdoferi. Moreover, CSF polymerase chain reaction (PCR) for CMV, EBV, HSV 1,2, *Mycoplasma pneumoniae*, and VZV was performed. Severe acute respiratory syndrome Coronavirus 2 (SARS-CoV-2) research was carried out in the years 2020 and 2021. A search for *Campylobacter Jejuni* in stool samples was carried out in all the subjects with AMAN.

In all subjects, the presence of immunoglobulin (IgG/IgM) antibodies to gangliosides GM1 and GQ1b was tested by using a conventional enzyme-linked immunosorbent assay. From 2019 to 2021, laboratory tests were implemented in the search for other antiganglioside antibodies (GM2-4; GD1a,b; GD2,3; GT1a,b; sulfatide, both IgM and IgG) and anti-myelin-associated glycoprotein IgM antibodies. The common blood test was finally analyzed (C-reactive protein, erythrocyte sedimentation rate, complete blood cell count, glucose, creatinine, and ALT/AST).

### Electrophysiological Examination

Electrophysiological examination for all the patients included at least four motor nerves (median, ulnar, deep peroneal, and tibial) and three sensory nerves (median, ulnar, and sural). In a large number of subjects, especially if there was the need to obtain further data to clarify the axonal or demyelinating nature of the polyneuropathy, the aforementioned nerves were examined bilaterally, adding also the study of the superficial peroneal and radial nerves. This eventuality occurred especially in patients in whom the examination was carried out very early with respect to the onset of symptoms (the electrical abnormalities may not be that prominently evident for definite diagnosis in the first week).

The electrophysiological examination included minimal F-wave, motor and sensory conduction velocities, distal motor latency, compound muscle action potential amplitude (CMAPa), and sensory nerve action potential amplitude (SNAPa). We used surface recording electrodes. A constant current stimulator delivered electrical stimuli through bipolar surface electrodes (2-cm inter-electrode distance, cathode distal). Stimulating ring electrode on the fingers (cathode at the first interphalangeal joint) was used for sensory neurography in the upper arms. The recording muscles included the abductor pollicis brevis, abductor digiti minim i, extensor digitorum brevis, and abductor hallucis brevis. Ulnar, median, and deep peroneal CMAPs were obtained in at least three stimulation points along the nerve course. CMAPa was calculated as the baseline-negative peak while SNAPa peak-to-peak. Distal motor latency was measured at the onset of CMAP.

Skin temperature was maintained at >32°C in an infrared lamp. Neurographic values that were at least 2 standard deviations (SDs) above or below the mean normative data in our laboratory were considered abnormal.

Acute inflammatory demyelinating polyneuropathy was diagnosed in patients with electrophysiological signs of demyelination in at least two motor nerves, such as: prolonged distal latency>125% of the upper limit of normal; motor velocity slowing <80% of the lower limit of normal; prolonged F-wave latency >120% of the upper limit of normal; recording of a conduction block, defined as proximal/distal CMAP amplitude ratio <50%; or abnormal temporal dispersion, defined as increased proximal CMAP duration by more than 15%. Axonal GBS was diagnosed in patients without evidence of demyelination who had decreased CMAP amplitudes below 80% of the lower limit of normal in at least two nerves. Overall, we used the electrophysiological criteria of Hadden et al. (15) and Uncini and Kubawara (16). Aware that in the case of node-paranodopathies some axonal GBS could be classified as demyelinating (for example, in the case with a reversible conduction failure), to properly distinguish the axonal from the demyelinating form, we considered the second electrophysiological study (16). Patients not fulfilling the criteria for AIDP or AMAN were classified as “equivocal”.

### Statistical Analysis

Categorical variables were described using frequencies and percentages while continuous variables by median and interquartile range or mean and SD.

The patients were further divided into two different groups according to the electrophysiological type and need of MV. Because most of the data were not normally distributed according to the Kolmogorov–Smirnov Test, we used the Mann–Whitney test to perform the statistical differences of continuous variables, while a chi-square test was used when comparing categorical variables. The level of statistical significance was set at *p* < 0.05. Multivariate logistic regression analysis was performed to find variables influencing the need for MV. After dichotomizing the “need for MV” into two dependent variables (presence/absence of need for MV = 0/1), a logistic regression analysis was performed by using the variables significantly associated with the need for MV (suitably dichotomized) as independent variables. All the tests were performed with SPSS, version 23 or Graphpad-Prism.

## Results

**Figure 1:**
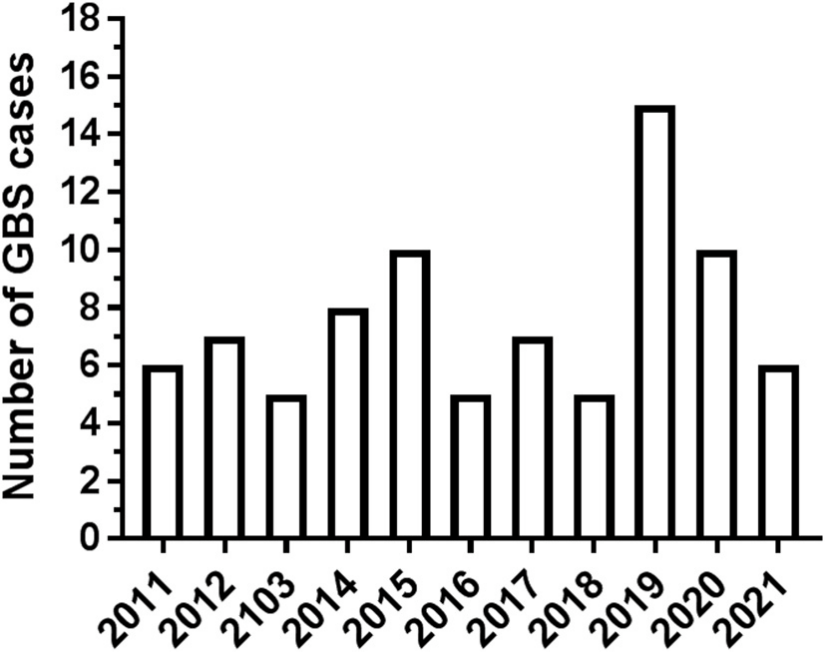
Number of Guillain-Barré syndrome (GBS) cases per year.

**Figure 2:**
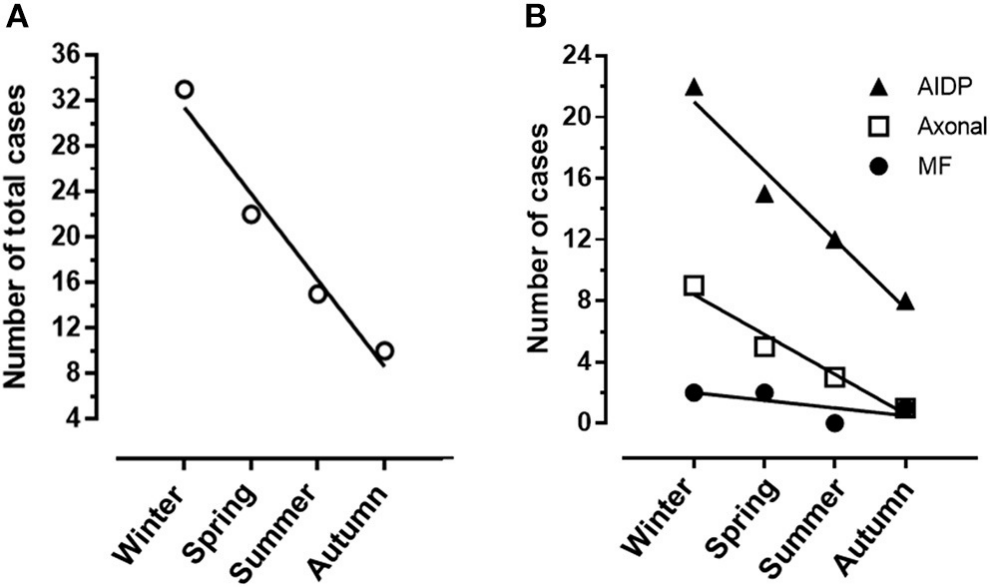
**(A)** Heterogeneity of Guillain-Barré syndrome (GBS) incidence among seasons with a significant cluster in winter months. **(B)** Different distribution of demyelinating (AIDP) and axonal GBS among seasons.

A total of 84 patients with GBS hospitalized in our Center in the decade 2011–2021 were considered for the analysis. In total, four subjects were not included because complete details were not available. Patient distribution over the 11 years of the study is shown in Figure 1. Hospitalization of patients with GBS showed a seasonal variation, with increased incidence in winter (see Figure 2A). However, when patients were grouped for the electrophysiological subtype, a seasonal clustering was seen only for AIDP (Figure 2B).

### Clinical, Laboratory, and Electrophysiological Features of the Patients

Table 1 reports clinical, laboratory, and electrophysiological features of the patients. The median (interquartile range [IQR]) age was 61 (45–71) years, the male/female ratio was 1.54 (33 women and 51 men). In total, two patients with a clinical and electrophysiological time course typical of a demyelinating GBS, had symptoms relapses after six–seven months from the GBS recovery; retrospectively, acute-onset chronic inflammatory demyelinating polyneuropathy was diagnosed. A patient with AMAN, 3 years later developed a multifocal motor neuropathy (17).

**Table 1:** Epidemiological, clinical, laboratory, and electrophysiological features of 84 patients with GBS.

**PRECEDING EVENTS**	**N. cases, percentage and electrophysiological type**
Gastroenteritis	15 tot (17.8%), 10 AIDP, 5 AMAN
Upper resp. Tract infection	9 tot (10.7%), 6 AIDP, 1 AMAN, 1 AMSAN, 1 MFS
Flu syndrome	22 tot (26.2%), 14 AIDP, 3 AMSAN, 2 AMAN, 1 MFS, 2 Eq
Urinary infection	2 tot (2.4%), 1 AIDP, 1 AMAN
Post Covid infection	1 tot (1.2%), 1 AMSAN
Vaccination^*^	2 tot (2.4%), 2 AIDP
Other^**^	2 tot (2.4%), 1 AIDP, 1 AMAN
None	31 tot (36.9%)
**IgM PRESENCE**	**N. cases and electrophysiological type**
HSV 1,2	1 AIDP
Mycoplasma pn.	1 AIDP
Mycoplasma pn + HSV 1,2	1 AMSAN
EBV	1 AIDP
CMV	4 AIDP
CMV + Mycoplasma pn.	1 AIDP
**CONCOMITANT INFECTION**	**N. cases and electrophysiological type**
CMV	5 AIDP
**ELECTROPHYSIOLOGICAL TYPE**	**N. cases and percentage**
AIDP	56 (66.6%)
AMSAN	9 (10.7)
AMAN	8 (9.5)
AMAN + AMSAN (Axonal)	17 (20.2)
MFS	5 (5.9.%)
Equivocal	6 (7.1%)
**PRESENTING SYMPTOMS**	**N. cases and percentage**
Typical^***^	8 tot (69%), 45 AIDP, 7 AMSAN, 5 AMAN, 1 MFS
Only motor symptom	21 tot (25%), 11 AIDP, 3 AMSAN, 3 AMAN, 4 MFS
Cranial nerve involvement	29 tot (34.5%); 17 AIDP, 4 AMSAN, 3 AMAN, 5 MFS
**GBS DISABILITY SCORE**	**Median (IQR)**
On admission/on discharge	AIDP 2 (2–3)/2 (1–3)
On admission/on discharge	AXONAL 3 (2–3)/2.5 (2–3)
On admission/on discharge	MFS 2.5 (2–3)/2 (2–2.75)
**LABORATORY DATA**	**N. cases and/or percentage**
CSF Protein (mg/dl)	59 (46–96.5)
ntibodies to gangliosides^****^	19 tot (22.6 %), 5 AIDP, 5 AMSAN, 5 AMAN, 4 MFS
Antibodies to GM1	2 IgG and IgM(2 AMSAN); 4 IgM (2 AIDP e 2 AMAN); 5 IgG (1 AIDP, 3 AMSAN, 2 AMAN)
Antibodies to GM2	1 IgM (AIDP); 1 IgG (AIDP)
Antibodies to GD1b and GQ1B	1 GD1b (AMSAN); 7 GQ1B (3 MF, 2 AIDP, 2 AMAN)
More altered laboratory data	ESR (36%), CRP (34.1%), white blood cell count (26.8%)

*AIDP, Acute inflammatory demyelinating polyneuropathy; AMAN, Acute motor axonal neuropathy; AMSAN, Acute motor sensory axonal neuropathy; CRP, C-reactive protein; EBV, Epstein-Barr virus; Eq, equivocal; ESR, Erythrocyte sedimentation rate; GBS, Guillain Barrè Syndrome; HSV, herpesvirus; IQR, interquartile range; MFS, Miller Fisher syndrome; n, number; pn, pneumoniae; tot, total number of all patients regardless of electrophysiological type*.

* *1 Astra Zeneca, 1 flu vaccine;*

** *meningioma and basal cell exeresis;*

*** *typical: distal paresthesia and hyposthenia;*

**** *two patients have antibodies to GQ1B and GM1 (IgG)*.

*The search for GM2-4; GD1a,b; GD2,3; GT1a,b; sulfatide and anti MAG antibodies was performed only in 21 subjects*.

A previous infective event in the 4 weeks before neurological onset was seen in 63.1% of patients (see Table 1). Most (about 70%) of the AIDP with viral prodrome, occurred during the winter. The median (IQR) of the hospital stay was 10 (8–14) days. For patients discharged to a rehabilitation center, hospital stay times have sometimes been lengthened while awaiting admission. The patients with axonal GBS had a longer hospital stay than those with AIDP even if the difference was not statistically significant. The median (IQR) time elapsed between symptom onset to hospital admission was 4 (2–8) days.

Most patients (69%) had typical symptomatology, that is tingling/hypoesthesia and weakness starting from the feet and spreading to the leg, and upper limb, 25% had pure motor form. Cranial nerve involvement occurred in 34.5% of GBS cases, with peripheral facial palsy being the most frequent one. Anti-ganglioside antibodies were detected in 22.6% of the cases.

In Table 2, the characteristics of the patients with AIDP with respect to patients with axonal forms (AMAN and AMANS) are shown. Significant differences between the axonal and myelinic forms of GBS concerned the presence of anti-ganglioside antibodies (more frequent in the axonal forms). We did not find any cases with reversible conduction failure.

**Table 2:** Comparison between patients with AIDP form and those with axonal (AMAN/AMSAN) form.

	**AIDP (n.56)**	**AXONAL (n.17)**	***p*** **-value**
Age (median, IQR)	61 (48–72)	62.5 (36.7–75)	n.s
Sex female/male	20/36	7/10	n.s
Gastroenteritis (n. cases)	10	5	n.s.
Flu Syndrome (n. cases)	13	5	n.s.
Upper respiratory infection (n. cases)	6	2	n.s.
IgM presence (n. cases)	7	2	n.s.
GBS score in /out (median, IQR)	2 (2–3)/2 (1–3)	3 (2–3)/2.5 (2–3)	n.s.
Cranial nerve involvement (n. cases)	17 (30.3%)	7 (41.2%)	n.s.
Typical presenting symptoms (n. cases)	45	12	n.s
Motor presenting symptoms (n. cases)	11	5	n.s.
Symptoms onset to admission (median, IQR)	4 (2–9)	4 (2.2–6.3)	n.s.
CSF Protein (median, IQR)	62 (46–113)	61 (48.7–76)	n.s.
Antibodies to gangliosides (n. cases)	5	11	*P* < 0.0001

*AIDP, Acute inflammatory demyelinating polyneuropathy;AMAN, Acute motor axonal neuropathy; AMSAN, Acute motor sensory axonal neuropathy; CFS, cerebrospinal fluid; GBS, Guillain Barrè Syndrome; IQR, interquartile range; n, number. P values were obtained by the chi-squared test for categorical variables and the Unpaired t test for continuous variables*.

In 9 subjects (10.8%), IgM presence for the virus was detected. In 5 subjects, a primary CMV infection was demonstrated by serum positive anti-CMV IgM, low avidity of the anti-CMV IgG and CMV–DNA presence. Laboratory tests revealed anti *Mycoplasma pneumoniae* IgM in 3 subjects, anti-HSV 1,2 IgM in 2, and anti-EBV IgM in 1 (Table 1). None of these 9 patients had anti-ganglioside antibodies, except in one case with IgM for Mycoplasma pneumonia and HSVs 1 and 2, in which antibodies against GD1b were found.

### Treatment

As to the treatment, 70 patients (83.4%) received intravenous immunoglobulin (IVIg) at conventional doses (2 g/kg), and 14 patients (16.6%) received four or five sessions of plasmapheresis. Plasmapheresis was not preferred due to its invasiveness, however, it was used in subjects with thrombotic diathesis. In the three severe GBS cases, plasmapheresis was combined with IVIg. In almost all patients, the treatment was started within 2 days from admission, without substantial side effects. No difference was noted in the effectiveness of treatment or improvement rate by the usage of either treatment. Subjects with concomitant CMV primary infection received Ganciclovir 5 mg/Kg/die I.V. until CMV–DNA disappeared from the plasma.

### Outcome

At hospital discharge, 26.2% of patients had mild symptoms, mainly sensory in nature, and were able to perform daily activities and to run, whereas 20 subjects (14.3%) were unable to walk even with the bilateral support. No patients died. There were no significant differences in clinical status between demyelinating and axonal forms.

In total, forty-one of the 84 patients were presented with mild disease (GBS disability scores 1 or 2), and 43 presented with moderate disease (GBS disability scores 3 or 4). At hospital discharge, of the 41 subjects with the mild disease on presentation, 3 lost the ability to walk independently and 3 were bedridden or chair bound. Of the 43 patients presenting with moderate disease, 9 could not stand, even with support (8 AIDP, 1 AMAN). At hospital discharge, elderly patients (i.e., subjects > 60 years) had higher GBS disability scores to respect to the other patients (i.e., subjects ≤ 60 years).

In total, seven (8.33%) patients required MV. Table 3 showed the characteristics of this group compared to all the others subjects. We found that patients requiring MV during hospitalization were older, had higher GBS disability scores, more frequent motor symptoms, dysautonomia, and cranial nerve involvement on admission to the hospital. Coexisting CMV infection was significantly associated with the requirement for subsequent MV. The multivariate logistic regression analysis is shown in Table 4 illustrates the *p*-values, β values, odds ratio, and 95% CI of each significant variable.

**Table 3:** Comparison between patients requiring mechanical ventilation (MV) and the others GBS subjects.

	**Patient requiring MV**	**Other patients**	***p*** **-value**
	**7 subjects (8.3%)**	**77 subjects (91.7%)**	
Age (median, IQR)	77 (54–86)	60 (45–69)	0.04
Sex female/male	3/4	30/47	n.s.
Symptoms onset at hospital admission (days; median, IQR)	3.8 (3–10)	4 (2–8)	n.s.
GBS disability score (median, IQR)	3.5 (2.2–4)/4 (3.2–4)	2 (2–3)/2 (1–3)	0.004/ <0.0001
Electrophysiological type (n. cases)	4 AIDP, 1 AMSAN, 1 MFS, 1 Eq	51 AIDP, 5 Eq, 9 AMSAN, 8 AMAN, 4 MFS	n.s.
Cranial nerve (n. cases, percentage)	6 (85.7%)	23 (29.9%)	0.0061
Concomitant CMV infection (n. cases, percentage)	2 (28.6%)	3 (3.9%)	0.0082
CSF Protein(median, IQR)	55 (42–63)	63 (46–110)	n.s.
Antibodies to gangliosides (n. cases, percentage)	1 (14.3%)	18 (23.4%)	n.s.
Motor presenting symptoms (n. cases, percentage)	5 (71.4%)	17 (22.1%)	0.0045
Dysautonomia^*^ (n. cases, percentage)	3 (42.8%)	7 (9.1%)	0.0083

*AIDP, Acute inflammatory demyelinating polyneuropathy; AMAN, Acute motor axonal neuropathy; AMSAN, Acute motor sensory axonal neuropathy; CMV, cytomegalovirus; CFS, cerebrospinal fluid; Eq, equivocal; GBS, Guillain Barrè Syndrome; IQR, interquartile range; no., number. P-values were obtained by the chi-squared test for categorical variables and the Unpaired t test for continuous variables*.

* *cardiac rhythm disturbances, hypertension/hypotension*.

**Table 4:** Multivariable logistic regression model for variables associated with ICU admission.

**Variables**	**β**	**OR**	**95% CI**		***p*** **-value**
Age	1.119	3.062	0.271	34.533	0.365
GBS disability score	2.085	8.042	0.556	116.428	0.126
Cranial nerve involvement	2.424	11.289	1.101	115.743	0.04
Concomitant CMV infection	2.572	13.096	0.622	275.8	0.098
Motor presenting symptoms	2.122	12.11	0.756	195.328	0.095
Dysautonomia	2.012	9.142	0.822	281.111	0.101

*Age was dichotomized according to the following criterion: >60 years = 1, ≤ 60 years = 0. The GBS disability score was dichotomized according to the following criterion: GBS score 1 or 2: 0; GBS 3-5:1. CI, confident interval; CMV, cytomegalovirus; GBS, Guillain–Barrè Syndrome; OR, odds ratio*.

Finally, in the GBS group requiring MV, 2 subjects had oncological pathologies and diabetes, and 2 pre-existing lung diseases. In the GBS group not requiring MV, only 3 subjects had oncological pathologies and 8 diabetes. In total, six subjects were treated with IVIg, 1 with plasmapheresis followed by IVIg. The symptomatic treatment of dysautonomia consisted of beta blockers, calcium channel blockers, and ACE inhibitors.

## Discussion

In the 11-year period of the current study, 84 patients with GBS were identified in our Center. In agreement with other studies, in the present cohort men were more frequently affected than women (18) and an increased incidence of GBS was observed in the winter periods. There is not a worldwide consensus on the seasonality of GBS, as higher rates seem to occur in winter in the colder regions and in spring in temperate areas (19). Subjects with concomitant CMV infection (see later) are an exception, since only 1 out of 5 seem to occur in the winter. The maximum number of GBS diagnosis during the decade was in 2019 (*n* = 15), without a clear increase during the SARS-CoV-2 pandemic (4 diagnoses in the last trimester of 2019, 10 in 2020, and 6 in 2021). This is in disagreement with some recent data reporting SARS-CoV-2 infection as the trigger of GBS (20, 21). Indeed, a recent study does not support the hypothesis of a significant link between SARS-CoV-2 infection and GBS (22). We believe that SARS-CoV-2, like all viruses, could induce GBS by dysregulation of the immune response; nevertheless, the incidence of GBS during the pandemic may not have been exceptionally high due to the lockdown measures reducing transmission of GBS inducing pathogens, such as respiratory viruses.

In according with Zhang et al. (23), elderly subjects had poor short-term prognosis at discharge.

### Preceding Infection/Vaccination

About 63% of the patients had previous infective events within 4 weeks before symptomatic onset. In agreement with the results of an Italian multicenter study, the most frequent prodromic events were flu-like syndrome and gastroenteritis (24). Interestingly, only one subject developed GBS after SARS-CoV-2 infection. Two subjects developed GBS a few weeks after vaccination, one with AstraZeneca COVID-19 vaccine and one with a flu vaccination. Differently from the data of the ITANG study (2), no significant effect of the 2011 H1N1 influenza pandemic or vaccination against it for GBS occurrence was observed.

In 9 subjects (10.7%), we found IgM against *Mycoplasma Pneumoniae*, HSV1,2, EBV, and CMV. In 5 subjects (5.9%), a definite concomitant CMV primary infection was demonstrated by the presence of anti-CMV IgM and IgG, CMV-IgM low avidity, and detection of CMV-DNA in plasma by the use of in-house PCR assays. Our percentage is higher than that reported by another Italian monocentric study (3.3% of cases) (11), and lower than that reported in a French GBS cohort (12.5% of cases) (25). CMV infection is considered to be the second most common infection preceding GBS (26) and these patients are younger, and often show AIDP type, facial nerve palsy, sensory loss, anti-GM2 IgM positivity, and severe course (25, 27). Indeed, in our CMV-GBS subjects, the median (IQR) age was 39 years, all had AIDP form, facial involvement, three had autonomic disturbances, such as cardiac arrhythmias and blood pressure fluctuations, and one required endotracheal intubation for ventilation support and another had respiratory failure requiring noninvasive ventilation. On admission, the GBS disability score of patients with CMV–GBS was not different from that of the other patients with GBS, while that at discharge was much higher (median 4). However, at 6 months follow-up, all subjects were able to walk without support. Antibodies against ganglioside GM2 are frequently present in the serum from patients with GBS with an antecedent infection with CMV (28–30); however, none of our CMV–GBS samples had this antibody positivity. To date, little is known about the physiopathology of CMV–GBS, and the roles of anti-ganglioside antibodies, cellular immune responses, and viral replication are not yet established. Our data seem to corroborate the assumption that is unlikely that CMV infection and anti-ganglioside GM2 antibodies are solely responsible, and an additional factor is required to elicit GBS (28).

### Epidemiology and Clinical Characteristics of Electrophysiological Subtypes

As expected from the epidemiological studies, AIDP was the most common subtype (66.6%), whereas the incidence of AMAN/AMSAN accounts for about 20% of the cases. The axonal subtype is slightly over-represented in our cohort, considering that for European countries the frequencies of axonal GBS had been established between 6 and 17% (31). The number of axonal subtypes was in line with that reported in a cohort of Rome (32), higher and lower than those reported in the populations of Ferrara and La Spezia, respectively (11, 12). The different geographical distribution of GBS types may lead to considering the role of a possible environmental factor.

We did not find differences in sex, age, presenting symptoms, preceding conditions, and cranial nerve involvement among the GBS subgroups. It was reported that age, sex, and clinical disability score at first medical examination do not differ between patients with AMAN and AIDP (33), and our study yielded similar findings. In contrast to other studies, we did not observe a relationship between previous gastroenteritis infection and axonal subtype. Furthermore, we did not see a greater occurrence of the cranial nerve involvement in the AIDP subtype (34, 35), with the only significant difference between AIDP and the axonal forms being the known association of the antibody ganglioside positivity with AMAN/AMSAN electrodiagnosis (34).

### Epidemiology and Characteristics of Patients Required Mechanical Ventilation

In GBS, respiratory failure result from impaired secretion clearance and respiratory muscle weakness and approximately 20–30% of patients with GBS requiring MV (5, 36).

In our cohort, the subjects admitted to the intensive care unit were 7 (8.33% of cases), 2 with orotracheal intubation, and 5 with non-invasive ventilation. We have compared their characteristics with those of non-ventilated patients. The chi-square test was applied to compare the clinical presentation of the two groups: we identified older age, higher GBS disability score, and motor symptoms presence at admission, dysautonomia, cranial nerve involvement, and concomitant CMV infection as factors significantly associated with the intensive care unit admission. The characteristics of patients who requested MV are quite aligned with the potential risk factors for MV described in the literature (6, 7). Furthermore, among the 7 patients, only 1 did not have remarkable pathologies. In the remaining cases, 2 had cancer, 2 had pre-existing lung disease, and 2 had concomitant CMV infection. This data suggest that the occurrence of GBS in association with other pathological conditions, could increase the possibility of MV needs. The finding concerning the association between coexisting CMV infections and the requirement for MV is not new (37). This relationship may be explained by the observation that CMV-related GBS subjects tend to have more severe demyelination than the other patients with GBS and the demyelinating lesions are often located in the nerves involved in respiration (37).

The incidence of MV in our study was 8.33 %, which was much lower than the values reported in the literature (up 30% of cases) (9), but close enough to that of the Ferrara cohort (13.7%) (12). The different percentages of GBS subjects requiring MV have been tentatively attributed to the distribution of the different subtypes of GBS in different countries. In particular, patients with demyelinating types more frequently have cranial nerve involvement, which is considered a predictor for MV (9, 31). In our sample, there was no significant predominance of AIDP among the subjects requiring MV. A possible explanation for our low percentages of GBS-ventilated subjects could be found in the fact that intravenous immunoglobulins and/or plasmapheresis treatment have been initiated usually at a very early stage immediately after a clinical diagnosis is established, which might hinder the progression of the disease course.

There are limitations to our study. First of all, our study used retrospective analysis and the prognosis was performed on the hospitalized patients and lacked follow-up observations, making it impossible to analyze the long-term prognosis of GBS. Another limitation is that the search for Campylobacter Jejuni was not performed in all the GBS cases and in some subjects the antigangliosides antibodies analysis was incomplete.

## Conclusion

The epidemiological and clinical characteristics of GBS in different countries are constantly evolving, especially in relation to environmental changes. This study provides clinical-epidemiological information of a Tuscan cohort. The results obtained in this study can provide new insights into this severe neurological disorder. Due to the retrospective nature of the study, it is obviously possible that some information may have been lost, however, due to multiple assessments over time, the organizational layout of the neurological units, and the sharp inclusion criteria adopted, we are confident that the sources of error were minimized.

## Data Availability Statement

The raw data supporting the conclusions of this article will be made available by the authors, without undue reservation.

## Ethics Statement

Ethical approval for the study was obtained from the Local Ethic Committee of AOUS of Siena.

## Author Contributions

FGin, AR, and ND contributed to conception of the study and wrote the first draft of the antiganglioside. DC, GC, SB, NV, and CB contributed to the research of the data of the patients. FS, LF, and AM contributed for the electrophysiological part. All authors contributed to the manuscript revision, read, and approved the submitted version.

## Conflict of Interest

The authors declare that the research was conducted in the absence of any commercial or financial relationships that could be construed as a potential conflict of interest.

## Publisher's Note

All claims expressed in this article are solely those of the authors and do not necessarily represent those of their affiliated organizations, or those of the publisher, the editors and the reviewers. Any product that may be evaluated in this article, or claim that may be made by its manufacturer, is not guaranteed or endorsed by the publisher.

## References

[1] McGrogan A Madle GC Seaman HE de Vries CS. The epidemiology of Guillain-Barré syndrome worldwide. A systematic literature review. Neuroepidemiology. (2009) 32:150–63. 10.1159/000184748 19088488

[2] Benedetti MD Pugliatti M D'Alessandro R Beghi E Chiò A Logroscino G. multicentric prospective incidence study of Guillain-Barré syndrome in Italy. The ITANG Study. Neuroepidemiology. (2015) 45:90–9. 10.1159/000438752 26329724

[3] Willison HJ Jacobs BC van Doorn PA. Guillain-Barré syndrome. Lancet. (2016) 388:717–27. 10.1016/S0140-6736(16)00339-1 26948435

[4] Doets AY Verboon C van den Berg B Harbo T Cornblath DR Willison HJ. IGOS Consortium. Regional variation of Guillain-Barré syndrome. Brain. (2018) 141:2866–77. 10.1093/brain/awy232 30247567

[5] van den Berg B Storm EF Garssen MJP Blomkwist-Markens PH Jacobs BC. Clinical outcome of Guillain-Barré syndrome after prolonged mechanical ventilation. J Neurol Neurosurg Psychiatry. (2018) 89: 949–54. 10.1136/jnnp-2018-317968 29627773

[6] Green C Baker T Subramaniam. A Predictors of respiratory failure in patients with Guillain-Barré syndrome: a systematic review and meta-analysis. Med J Aust. (2018) 208:181–8. 10.5694/mja17.00552 29490222

[7] Wen P. Wang L. Liu H Gong L Ji H Wu H Chu W. Risk factors for the severity of Guillain-Barré syndrome and predictors of short-term prognosis of severe Guillain-Barré syndrome. Sci Rep. (2021) 11:11578. 10.1038/s41598-021-91132-3 34079013 PMC8172857

[8] Wu X Li C Zhang B Shen D Li T Liu K. Predictors for mechanical ventilation and short-term prognosis in patients with Guillain-Barre syndrome. Crit Care. (2015) 19:310. 10.1186/s13054-015-1037-z 26330143 PMC4557605

[9] Kobori S Kubo T Otani M Muramatsu K Fujino Y Adachi H. Coexisting infectious diseases on admission as a risk factor for mechanical ventilation in patients with Guillain-Barré syndrome. J Epidemiol. (2017) 27:311–6. 10.1016/j.je.2016.07.003 28283417 PMC5498408

[10] Jacobs BC Rothbarth PH van der Meché FG Herbrink P Schmitz PI de Klerk MA. The spectrum of antecedent infections in Guillain-Barré syndrome: a case-control study. Neurology. (1998) 51:1110–5. 10.1212/WNL.51.4.1110 9781538

[11] Benedetti L Briani C Beronio A Massa F Giorli E Sani C. Increased incidence of axonal Guillain-Barré syndrome in La Spezia area of Italy: A 13-year follow up study. J PeripherNerv Syst. (2019) 24:80–6. 10.1111/jns.12292 30421471

[12] Granieri E Andreasi NG De Martin P Govoni V Castellazzi M Cesnik E. Incidence study of Guillain-Barré syndrome in the province of Ferrara, Northern Italy, between 2003 and 2017. A 40-year follow-up. Neurol Sci. (2019) 40:603–9. 10.1007/s10072-018-3688-4 30617450

[13] Fokke C van den Berg B Drenthen J Walgaard C van Doorn PA Jacobs BC. Diagnosis of Guillain-Barré syndrome and validation of Brighton criteria. Brain. (2014) 137:33–43. 10.1093/brain/awt285 24163275

[14] Hughes RA Newsom-Davis JM Perkin GD Pierce JM. Controlled trial prednisolone in acute polyneuropathy. Lancet. (1978) 2:750–3. 10.1016/S0140-6736(78)92644-2 80682

[15] Hadden RDM Cornblath DR Hughes RAC. Electrophysiological classification of Guillain-Barré Syndrome: Clinical associations and outcome. Ann Neurol. (1998) 44:780–8. 10.1002/ana.410440512 9818934

[16] Uncini A Kuwabara S. Electrodiagnostic criteria for Guillain-Barré syndrome: a critical revision and the need for an update. Clin Neurophysiol. (2012) 123:1487–95. 10.1016/j.clinph.2012.01.025 22480600

[17] Bocci S Giannini F Volpi N Rossi A Ginanneschi F. Multifocal motor neuropathy occurring after acute motor axonal neuropathy: two stages of the same disease? Neurol Sci. (2022) 43:1463–5. 10.1007/s10072-021-05725-x 34800200

[18] Sejvar JJ Baughman AL Wise M Morgan OW. Population incidence of Guillain-Barré syndrome: a systematic review and meta-analysis. Neuroepidemiology. (2011) 36:123–33. 10.1159/000324710 21422765 PMC5703046

[19] Chroni E Papapetropoulos S Gioldasis G Ellul J Diamadopoulos N Papapetropoulos T. Guillain-Barré syndrome in Greece: seasonality and other clinico-epidemiological features. Eur J Neurol. (2004) 11:383–8. 10.1111/j.1468-1331.2004.00799.x 15171734

[20] Filosto M Cotti Piccinelli S Gazzina S Foresti C Frigeni B Servalli MC. Guillain-Barré syndrome and COVID-19: an observational multicentre study from two Italian hotspot regions. J Neurol Neurosurg Psychiatry. (2021) 92:751–6. 10.1136/jnnp-2020-324837 33158914

[21] Agosti E Giorgianni A D'Amore F Vinacci G Balbi S Locatelli D. Is Guillain-Barrè syndrome triggered by SARS-CoV-2? Case report and literature review. Neurol Sci. (2021) 42:607–12. 10.1007/s10072-020-04553-9 32643136 PMC7343406

[22] Keddie S Pakpoor J Mousele C Pipis M Machado PM Foster M. Epidemiological and cohort study finds no association between COVID-19 and Guillain-Barré syndrome. Brain. (2021) 144:682–93. 10.1093/brain/awaa433 33313649 PMC7799186

[23] Zhang B Wu X Shen D Li T Li C Mao M. The clinical characteristics and short-term prognosis in elderly patients with Guillain-Barré syndrome. Medicine (Baltimore). (2017) 96:e5848. 10.1097/MD.0000000000005848 28072747 PMC5228707

[24] Guillain-Barre Syndrome Study Group. Guillain-Barre syndrome: an Italian multicentre case control study. Guillain-Barre Syndrome Study Group. Neurol Sci. (2000) 21:229–34. 10.1007/s100720070081 11214662

[25] Orlikowski D Porcher R Sivadon-Tardy V Quincampoix JC Raphaël JC Durand MC. Guillain-Barré syndrome following primary cytomegalovirus infection: a prospective cohort study. Clin Infect Dis. (2011) 52:837–44. 10.1093/cid/cir074 21427390

[26] Hughes RA Hadden RD Gregson NA Smith KJ. Pathogenesis of Guillain-Barré syndrome. J Neuroimmunol. (1999) 100:74–97. 10.1016/S0165-5728(99)00195-2 10695718

[27] Leung J Sejvar JJ Soares J Lanzieri TM. Guillain-Barré syndrome and antecedent cytomegalovirus infection, USA 2009–2015. Neurol Sci. (2020) 41:885–91. 10.1007/s10072-019-04156-z 31828680 PMC7501740

[28] Khalili-Shirazi A Gregson N Gray I Rees J Winer J Hughes R. Antiganglioside antibodies in Guillain-Barre' syndrome after a recent cytomegalovirus infection. J Neurol Neurosurg Psychiatry. (1999) 66:376–9. 10.1136/jnnp.66.3.376 10084538 PMC1736267

[29] Ang CW Jacobs BC Brandenburg AH Laman JD van der Meché FG Osterhaus AD. Cross-reactive antibodies against GM2 and CMV-infected fibroblasts in Guillain-Barre'syndrome. Neurology. (2000) 54:1453–8. 10.1212/WNL.54.7.1453 10751257

[30] Yuki N. Infectious origins of, and molecular mimicry in, Guillain-Barre' and Fisher syndromes. Lancet Infect Dis. (2001) 1:29–37. 10.1016/S1473-3099(01)00019-6 11871407

[31] Kuwabara S Yuki N. Axonal Guillain-Barré syndrome: concepts and controversies. Lancet Neurol. (2013) 12:1180–8. 10.1016/S1474-4422(13)70215-1 24229616

[32] Luigetti M Servidei S Modoni A Rossini PM Sabatelli M Lo Monaco M. Admission neurophysiological abnormalities in Guillain-Barré syndrome: A single-center experience. Clin Neurol Neurosurg. (2015) 135:6–10. 10.1016/j.clineuro.2015.05.001 26001516

[33] Hiraga A Mori M Ogawara K Hattori T Kuwabara S. Differences in patterns of progression in demyelinating and axonal Guillain-Barré syndromes. Neurology. (2003) 61:471–4. 10.1212/01.WNL.0000081231.08914.A1 12939419

[34] Sekiguchi Y Uncini A Yuki N Misawa S Notturno F Nasu S. Antiganglioside antibodies are associated with axonal Guillain-Barré syndrome: a Japanese-Italian collaborative study. J Neurol Neurosurg Psychiatry. (2012) 83:23–8. 10.1136/jnnp-2011-300309 22010183

[35] Tian J Cao C Li T Zhang K Li P Liu Y. Electrophysiological subtypes and prognostic factors of Guillain-Barre Syndrome in Northern China. Front Neurol. (2019) 10:714. 10.3389/fneur.2019.00714 31333568 PMC6614537

[36] Yuki N Hartung HP. Guillain-Barré syndrome. N Engl J Med. (2012) 366:2294–304. 10.1056/NEJMra1114525 22694000

[37] Visser LH van der Meché FG Meulstee J Rothbarth PP Jacobs BC Schmitz PI. Cytomegalovirus infection and Guillain-Barré syndrome: the clinical, electrophysiologic, and prognostic features. Dutch Guillain-Barré Study Group Neurol. (1996) 47:668–73. 10.1212/WNL.47.3.668 8797462

